# The role of middle managers in tobacco control after a national smoke-free hospital campus ban

**DOI:** 10.1186/s12913-016-1764-0

**Published:** 2016-09-23

**Authors:** Cristina Martínez, Montse Ballbè, Miquel Vilardell, Marcela Fu, Esteve Fernández

**Affiliations:** 1 Tobacco Control Unit, Cancer Control and Prevention Programme, Institut Català d’Oncologia-ICO, Av. Granvia de L’Hospitalet 199-203, 08908 L’Hospitalet de Llobregat Barcelona, Spain; 2 Cancer Control and Prevention Group, Institut d’Investigació Biomèdica de Bellvitge-IDIBELL, Av. Granvia de L’Hospitalet 199-203, 08908 L’Hospitalet de Llobregat Barcelona, Spain; 3 Medicine and Health Sciences School, Universitat Internacional de Catalunya, C. Josep Trueta s/n, 08915 Sant Cugat del Valles Barcelona, Spain; 4 Addictions Unit, Institute of Neurosciences, Hospital Clínic de Barcelona, C. Villarroel 170, 08036 Barcelona, Spain; 5 Department of Clinical Sciences, School of Medicine, Universitat de Barcelona, C. Feixa llarga s/n, 08907 L’Hospitalet del Llobregat Barcelona, Spain; 6 Department of Prevention, Hospital de Vic, Vic, Barcelona, Spain; 7 Tobacco Control Unit, Institut Català d’Oncologia, Av. Gran Via de L’Hospitalet, 199-203, E-08908 L’Hospitalet de Llobregat Barcelona, Spain

**Keywords:** Smoke-free policies, Hospital, Sociometric analysis, Mixed methods, Managers

## Abstract

**Background:**

Much of the recent health services research on tobacco control implementation has explored general views and perceptions of health professionals and has rarely taken into account middle management’s perspectives. We state that middle managers may facilitate the implementation of smoke-free campus bans and thereby improve their effectiveness. The aim of this study was to assess middle managers’ behaviors to enforce a new national smoke-free hospital campus ban, to evaluate their perceptions of the level of compliance of the new regulation, and to explore their attitudes towards how smoking affects the work environment.

**Methods:**

We used a cross-sectional survey, conducted online to evaluate middle managers of a general hospital in Catalonia, Spain. Close-ended and open-ended questions were included. Results were analyzed by using quantitative and qualitative methods. The managers’ open opinions to the proposed topics were assessed using UCINET, and a graph was generated in NetDraw.

**Results:**

Sixty-three of the invited managers (78.7 %) participated in the survey. 87.2 % of them agreed that the hospital complied with the smoke-free campus ban and 79.0 % agreed that managers have an important role in enforcing the ban. They also perceived that smoking disturbs the dynamics of work, is a cause of conflict between smokers and non-smokers, and harms both the professional and the organization images. However, 96.8 % of respondents have never given out fines or similar measures and their active role in reminding others of the policy was limited; in addition, 68.2 % considered that hospitals should provide tobacco cessation treatments. Smoker middle managers were more likely than non-smokers to perceive that smoking has little impact on work.

**Conclusions:**

Middle managers play a limited role in controlling tobacco consumption; smokers are less prone to think that smoking disturbs work dynamics than non-smokers. Tailored training and clear proceedings for middle managers could encourage more active roles.

## Background

Health care organizations frequently face new challenges such as the implementation of policy innovations. Over the last decades, many health care services have enforced smoke-free policies by adopting well-established models — such as the Joint Commission on Accreditation of Healthcare Organizations in the United States [1], the Ottawa model in Canada [2], and the ENSH-Global Network for Tobacco-free Hospitals [3] — in addition to smoke-free legislation [4].

Smoke-free policies within hospitals are responsible for several public health benefits, such as (a) protecting non-smokers from second-hand smoke (SHS) [5]; (b) increasing the number of quit attempts and reducing the prevalence of smoking [6]; and (c) promoting tobacco cessation activities and establishing a role models [2, 7–9].

However, successful policy implementation requires a great organizational effort [10]. Two out of three organization innovations fail [10], mainly due to low commitment and lack of planning [11]. Research has identified that effective implementation depends on the commitment of top managers, internal communication of the project, the organization’s support activities — i.e. training, resources, presentations, and meetings —, and the willingness of individuals, mainly health care providers to implement these activities on the frontlines [12, 13]. Middle managers are in a unique position to improve implementation because they can potentially influence the decisions of top management as well as the performance of frontline employees [13]. In addition, a team work approach with a pivotal middle manager has become popular in healthcare organizations; thus, there is increasing potential for middle managers to influence the implementation process [13].

Rogers’ diffusion of innovation model points out the importance of fitting the innovation with the beliefs of those responsible for the implementation [14]. In this sense, both top managers, who direct and decide the innovations, and middle managers, who supervise frontline employees and oversee the implementation of the innovation, should believe in the potential benefits of the new innovation. Middle managers may perform both clinical and managerial tasks and play a relevant role in the success of the implementation [10]. In particular, middle managers make sure that hospital staff members are performing their designated duties and maintain open lines of communication among departments. Therefore, the behaviors, perceptions, and attitudes of middle managers significantly impact the implementation of policies; thus, there is a need for organizational responses to the local work environment [15]. The gap between evidence and effective care and practices should be narrowed [16].

Much of the recent health services research on tobacco control implementation has explored the general attitude and perception of health professionals [9, 17–19]. It has rarely taken into account the perspectives of managers or middle management. To our knowledge, only one quantitative study delved into the top manager’s standpoint, but the views of middle managers were not explored [15].

The Theory of Planned Behavior proposed by Ajzen states that humans are rational analyzers of the situation and one’s intention is actually what mediates attitude and behavior. Therefore, both attitudes and perceptions can determine behavior [20]. So, exploring these three elements may reveal how middle managers can help implement a new smoke-free hospital campus policy. We hypothesized that middle managers can facilitate the implementation of an innovation (e.g. smoke-free campus policy) and, thereby, improve its effectiveness. In Spain, a new smoke-free legislation concerning all hospital premises (including outdoor areas) was passed in January 2011.

Therefore, the aims of this study were to assess middle managers’ *behaviors* used to enforce the new smoke-free campus policy, to evaluate their *perceptions* of compliance of the new regulation (how smoking affects work dynamics, hygiene, professional relationships, patient recovery, etc.), and explore their *attitudes* towards the role of health professionals in tobacco control. The results were analyzed according to the managers’ sex, profession, and smoking status.

## Methods

This study capitalized on a hospital that has pursued a 12-year comprehensive tobacco control strategy and was influenced by the recent passage of a national smoke-free campus ban in hospitals in January 2011. The data were obtained using an internet-based cross-sectional survey conducted from March to July 2012.

### Participants

Study participants were middle managers working in any of the departments of the “Hospital de Vic”, a hospital consortium located in Catalonia (in the North East of Spain). This is an acute reference hospital with 364 beds and 1184 workers. At the time of the study, there were 80 middle managers working for the whole organization and they were all invited to voluntarily participate in the survey.

### Survey instrument

We developed an *ad hoc* online questionnaire composed of 50 items to collect information regarding middle managers’ characteristics, behaviors, perceptions, and attitudes towards tobacco control. Policy evaluation items were developed by an *ad hoc* working group for this study and an expert group reviewed the content validity. The questionnaire (available upon request from the author) was posted on GoogleDocs using the account of the Tobacco Control Unit at the Catalan Institute of Oncology. The questions assessed the degree to which middle managers agreed or disagreed with some statements regarding the following three dimensions:

- *Behaviors:* actions how middle managers use to control tobacco in several situations, such as reminding of the policy, providing smoking cessation counselling, enforcing the policy, and so on. We included the following set of questions:
  (a.1) How often they have reminded workers, patients, and/or visitors of the smoke-free policy in the last month?
  (a.2) How often they have recommended quitting smoking to workers and/or patients in the last month?
  (a.3) Have they had working conflicts because of tobacco use with workers and/or patients in the last month?
  (a.4) Have they handed out fines/measures due to tobacco use to workers, patients, and/or visitors since the smoke-free campus ban was implemented (the 2^nd^ of January 2011)?
  (a.5) Have they had arguments related to tobacco consumption with workers, patients, and/or visitors since the smoke-free campus ban was implemented (the 2^nd^ of January 2011)?
- *Perceptions:* How smoking affects work dynamics, hygiene, professional relationships, patient recovery, and so on?
- *Attitudes*: Values and opinions on the role of health professionals, managers, and hospitals on tobacco control.

Responses to the questions related to (a) behaviors were measured on a five point scale where 0 = “never” and 5 = “five or more times/very frequently”. Responses to the questions on (b) perceptions and (c) attitudes were appraised on an 11 point discrete analogue scale where 0 = “total disagreement” and 10 = “total agreement”. Responses were recalculated into a qualitative variable to simplify overall interpretation (disagree = 0–4, neither agree nor disagree = 5; agree = 6– 10). Open-ended questions were also included in all situations and the statements posted.

The main independent variables included sex, profession, professional role, and smoking status. Profession was classified as health professionals (doctors, nurses, and others), or non-health professionals (administration, financial managers and others). We also asked about their professional role (clinician, non-clinician, or both). Smoking status was classified as daily smokers (currently smoking at least one cigarette/day), occasional smokers (currently smoking < 1 cigarette/day), former smokers (not smoking for 6 months or longer), and never smokers [21].

The questionnaire was piloted among five middle managers from the same hospital, who provided some recommendations and these results were not included in the final analysis. The study protocol obtained the approval of the Ethical Committee of the “Hospital de Vic” (reference: PR23/13). To increase the participant response, the link to the questionnaire was sent by e-mail explaining the study aims and asking for participation. The email briefly explained the overall aim of the survey and emphasized the voluntary nature of participation and the warranty of anonymity. Potential participants were e-mailed a maximum of five times to inquire about participation in the study, during the five months data were collected. Participants’ consent was implied when subjects completed and returned the questionnaire (as approved by the ethics committee).

### Analysis

We used a quantitative and a qualitative approach for the analysis. For *quantitative data*, we first performed a descriptive analysis of the main variables by calculating the frequencies and means (with standard deviations). Medians and interquartile ranges (IQR) were also computed due to the skewed distribution of data. We compared the managers’ perceptions and attitudes by sex, profession, and smoking status by means of Mann Whitney U non-parametric test. Statistical significance was set at 5 %. The data were computed using SPSS version 21.0. The *qualitative data* were drawn from open-ended questions on the different topics included in the survey. The content analysis was used to select and organize significant information into coded responses and to make comparisons between types of respondents (by sex, professional role, smoking status). Two researchers independently coded the data and discussed the coding. The information was classified according to some given topics. After this classification, we used sociometric techniques to classify information [22]. Briefly, central nodes (questions raised by the investigators) were matched to nodes nominated by managers (answers) using a matrix. The term network was depicted using the UCINET®’s tool Netdraw. In the figure, question nodes were colored in orange and answer nodes in green. The strength of the match, which denotes the number of repetitions established with the same question-answer node, is represented by the thickness of the interconnecting lines (the stronger the connection, the thicker the interconnection line between nodes).

This type of analysis provides an optimal match between issues that middle managers related to the topics suggested by the investigators. The matching provides a map of interrelated topics (or nodes). When a question topic (identified as an orange node) matches several topics (each one identified as a green node), that means that this issue was derived from different points of view of the responders. In the sociometric representation, the repetition of the match is denoted by its strength, but demonstrates the variability of the responders’ views/solutions/comments to the same problem.

## Results

The final sample included 63 respondents out of 80 (78.7 %) middle managers. From these, 25 (39.7 %) were doctors, 19 (30.2 %) nurses, 17 (27.0 %) administrative managers, and 2 (3.1 %) belonged to other professional groups. Approximately half of the respondents were women and the average age was 50.8 years old (SD: 6.3). The sample had an average professional experience of 26.5 years (SD: 7.0), and an average of 10.7 years (SD: 7.9) in the same managerial position at the time of the interview. By smoking status, 16.2 % were current smokers (daily or occasionally), 41.9 % former smokers, and 41.9 % never smokers.

### Middle managers’ behaviors for enforcing a smoke-free campus policy after the new law

Most respondents reported that they have never reminded workers, patients, and visitors of the smoke-free policy in the last month; while, only 58.7 % reported they never had a working conflict because of worker’s tobacco use and 87.1 % reported not having a conflict due to patient’s smoking in the last month (Table 1). Health professional middle managers (nurses, doctors) recommended workers quit smoking more often than non-health middle managers (administration and financial managers, among others) (Table 1).

**Table 1:** Middle managers’ attitudes about controlling tobacco in the hospital^a^

	Never		
	n	%	95%CI
How often have you reminded others about the policy in the last month?			
Workers	47	74.6	62.6-83.7
Patients	38	61.3	49.5-72.8
Visitors	60	94.7	86.9-98.3
How often have you recommended quitting smoking in the last month?			
Health professionals	44		
To workers	21	48.8	37.3-61.2
To patients	42	95.2	86.9-98.4
Non-health professionals	19		
To workers	13	70.0	57.6-79.7
To patients	13	70.0	57.6-79.7
How often have you had working conflicts because of tobacco use in the last month?			
Use by workers	37	58.7	46.4-70.0
Use by patients	55	87.1	76.9-93.4
How often have you had to give out fines/measures because of tobacco use since the smoke-free campus ban (2^nd^ January 2011)?			
To workers	59	96.8	89.1-99.1
To patients	46	76.3	64.4-85.0
To visitors	50	79.7	67.8-87.5
How often have you had arguments related to tobacco consumption since the smoke-free campus ban (2^nd^ January 2011)?			
With workers	55	87.1	76.9-93.4
With patients	46	76.3	64.4-85.0
With visitors	40	65.5	52.7-75.7

^a^ In some cases there are missing values

In addition, 76.3 % of middle managers never handed out fines or measures to patients, 79.7 % to visitors, and 96.8 % to workers, showing statistically significant differences among middle managers’ behavior when they deal with workers or patients. In addition, 65.5 % of middle managers reported never having arguments with visitors and 76.3 % with patients during the last month due to their tobacco consumption (Table 1).

### Middle managers’ perceptions of how smoking affects the work environment

The managers’ perceptions on how smoking affects the dynamics of the hospital, their relationship with smokers, their attitudes towards tobacco control, and their opinion on the role of hospitals and themselves in tobacco control are summarized in Table 2. The majority of middle managers agreed that workers’ smoking behaviors disturb the work dynamics (median = 8.0; IQR = 7.0–10.0), and are a cause of conflict between smokers and non-smokers (median = 8.0; IQR = 5.0–9.0). However, women and non-smokers were more likely to consider that smoking generates conflicts between smokers and non-smokers, being statistically significant in comparison to men and smokers respectively (see Table 3). They also agreed that smoking employees damage the image of health professionals (median = 10.0; IQR = 8.0–10.0), and their smoking habits do not adhere to hygienic standards (median = 8.0; IQR = 5.0–10.0) (Table 2). Only 37.1 % (median = 5.0, IQR = 1.0–7.0) of middle managers agreed that smokers neglect their duties more, with this opinion being higher among non-smokers than among smokers (*p* = <0.05) (Table 3). Furthermore, the vast majority (90.2 %) agreed that health professionals should set an example and not smoke. Finally, middle managers highly agreed with all the statements on tobacco control policies (>60 %) (Table 2).

**Table 2:** Middle managers’ attitudes, aptitudes, and perceptions on tobacco control^a^

	Disagree (Score 0–4)		No agreement No disagreement (Score 5)		Agreement (Score 6–10)		
	n	%	n	%	n	%	Median (IQR)
Managers’ perceptions of how workers’ smoking behaviors affect work							
Disturbs the work team dynamic	4	6.4	6	9.7	52	83.9	8.0 (7.0-10.0)
Generates conflicts between smokers and non-smokers	10	16.4	12	19.7	39	63.9	8.0 (5.0-9.0)
I have a more tense relationship with workers who smoke	48	76.2	9	14.3	6	9.5	1.0 (0.0-4.0)
Smokers slack off from their duties more often than non-smokers	27	43.5	12	19.4	24	37.1	5.0 (1.0-7.0)
Harms the health professional image	2	3.2	3	4.8	58	92.0	10.0 (8.0-10.0)
Threatens hygiene at the hospital	7	11.1	10	15.9	46	73.0	8.0 (5.0-10.0)
Health professionals should set an example and not smoke	3	4.9	3	4.9	55	90.2	9.0 (8.0-10.0)
Managers’ perceptions of how patients’ smoking behaviors affect work							
Disturbs the work team dynamic	8	13.8	12	19.0	41	67.2	7.0 (5.0-9.2)
Generates conflicts between workers and other patients	8	14.1	10	15.8	43	70.1	7.0 (5.0-9.0)
I have a more tense relationship with smoker patients	38	61.4	14	22.8	9	15.8	2.0 (0.0-5.0)
Smoker patients are more confrontational	13	21.5	23	35.7	27	42.8	5.0 (4.5-7.0)
Threatens the security of the organization	2	3.4	4	6.8	71	89.8	9.0 (8.0-10.0)
Affects his/her recovery	0	0.0	4	6.8	59	93.2	8.5 (8.0-10.0)
Managers’ attitudes towards tobacco control							
All health professionals should know how to assist smokers to quit	8	12.9	10	14.5	45	72.6	8.0 (5.0-10.0)
Managers have an important role in enforcing the smoke-free policy	6	9.5	7	11.1	50	79.4	9.0 (7.0-10.0)
Managers should work towards compliance of the law	3	4.8	5	7.9	55	87.3	9.0 (7.0-10.0)
Managers should foster smoking cessation among smoker patients	5	8.8	12	19.3	45	71.9	8.0 (5.0-9.0)
Hospitals should be role model organizations for controlling tobacco	1	1.6	1	1.6	61	96.8	9.0 (8.0-10.0)
Hospital top managers should allocate resources for tobacco control	7	11.3	16	25.8	40	62.9	8.0 (5.0-9.0)
Hospitals should provide tobacco cessation treatment for smoker patients	9	14.3	11	17.5	43	68.2	8.0 (5.0-9.0)
Hospitalization is a good time to quit smoking	6	9.5	7	11.1	50	79.4	8.0 (7.0-10.0)
This hospital complies with the smoke-free campus ban	5	8.0	3.0	4.8	53	87.2	8.0 (8.0-9.0)

^a^ In some cases there are missing values

**Table 3:** Middle managers’ attitudes, aptitudes, and perceptions towards tobacco control in the organization according to sex, professional group, and smoking status

	Sex			Professional group			Smoking status		
	Men *n* = 31	Women *n* = 32		Health workers^a^ *n* = 44	Non-health workers^b^ *n* = 19		Smoker^c^ *n* = 10	Non-smoker^d^ *n* = 53	
	median	median	p*	median	median	p*	median	median	p*
Managers’ perceptions of how workers’ smoking behaviors affect work									
Disturbs the work team dynamic	8.0	8.0	ns	8.0	9.5	ns	9.0	8.0	ns
Generates conflicts between smokers and non-smokers	6.0	8.0	<0.05	7.0	8.0	ns	5.0	8.0	<0.05
I have a more tense relationship with smoker workers	1.0	1.0	ns	1.0	1.5	ns	0.0	0.05	ns
Smokers slack off from their duties more often than non-smokers	4.0	5.0	ns	5.0	5.0	ns	1.0	5.0	<0.05
Harms health professional image	9.0	10.0	ns	10.0	9.0	ns	9.0	10.0	ns
Threatens hygiene conditions	8.0	8.0	ns	8.0	8.0	ns	7.5	8.0	ns
Health professionals should set an example and not smoke	9.0	9.5	ns	9.0	9.5	ns	7.0	10.0	<0.05
Managers’ attitudes towards tobacco control									
All health professionals should know how to assist smokers to quit	7.0	8.0	ns	8.0	8.0	ns	7.5	8.0	ns
Managers have an important role in enforcing the smoke-free policy	8.0	9.0	ns	8.0	9.0	ns	8.5	9.0	ns
Managers should work to ensure compliance of the law	8.5	9.0	ns	8.5	8.0	ns	8.0	9.0	ns
Managers should foster smoking cessation among smoker patients	7.0	8.0	ns	8.0	8.0	ns	7.5	8.0	ns
Hospitals should be role model organizations for controlling tobacco	9.0	9.0	ns	9.0	9.0	ns	9.0	9.0	ns
Hospital top managers should allocate resources for tobacco control	7.0	7.0	ns	7.0	5.5	ns	7.5	7.0	ns
Hospitals should provide tobacco cessation treatment for smoker patients	7.0	8.0	ns	8.0	8.0	ns	9.0	7.0	<0.05
Hospitalization is a good time to quit smoking	8.0	8.5	ns	8.0	8.5	ns	7.5	8.5	ns
This hospital complies with the smoke-free campus ban	8.0	8.0	ns	9.0	8.0	ns	8.0	8.0	ns

*ns* non-significant

*Mann–Whitney *U* test

^a^Non-health workers: administrative, financial, and other managers

^b^Health workers: doctors and nurses

^c^Smoker: daily and occasional smokers

^d^Non-smokers: former smokers and never smokers

Regarding smoking patients, middle managers considered that their smoking also disturbs the work dynamic, but less than smoking workers do (median = 7.0; IQR = 5.0–9.2, see Table 2).

#### Perceptions of the level of compliance with the smoke-free campus ban

Only 8.0 % of the respondents disagreed that the hospital complied with the smoke-free campus policy. More than 79.0 % agreed that managers have an important role in enforcing the smoke-free ban and that they should work towards its compliance (Table 2). In addition, 72.0 % considered that middle managers should have an active role in fostering smoking cessation among smoking patients.

### Middle managers’ attitudes towards the new smoke-free campus policy

Overall, 96.8 % of middle managers agreed that hospitals were role models of exemplary organizations (Table 2). About 73.0 % (median = 8.0, IQR = 5.0–10.0) of middle managers agreed that health providers should know how to assist smokers, 68.2 % considered that hospitals should provide tobacco cessation treatments, and 62.9 % considered that it is one of the duties of top managers to allocate resources for tobacco control (Table 2).

### Managers’ open responses and comments

Respondents had the opportunity to provide their personal opinion about the main topics included and not included in the survey related to tobacco control in the hospital. Overall, 113 open comments were given by 32 professionals. The topics more frequently raised were the compliance with the smoke-free campus ban, tobacco cessation, the role model, and the image of health professional smokers. The most relevant quotes on each of the mentioned topics and the number of comments obtained for each topic are summarized in Table 4.

**Table 4:** Illustrative quotes when referring to how tobacco consumption impacts the hospital

Image (13 comments)
1: “Seeing the health workers smoking (in their suits) outside the campus does not project a good image. Health professionals should be examples in following healthy lifestyles” (Woman, nurse, non-clinician, former smoker)
2: “Health professionals who smoke at the entrances give a bad image”(Woman, doctor, both^a^, never smoker)
3: “Butts around the hospital area give the impression that the hospital is a leisure area” (Man, nurse, both^a^, smoker)
Role model (7 comments)
4: “Patients should think that if health professionals have not stopped maybe it is not so important to quit” (Woman, doctor, both^a^, former smoker)
5: “As an organization and professional group we should not smoke ourselves” (Man, nurse, both^a^, former smoker).
6: “Everyone has the right to do what she/he wants out of the work area. But in the hospital it should be forbidden to smoke and to go smoke outside” (Man, nurse, both^a^, smoker)
Slack off (10 comments)
7: “Smoker workers need to go out of their workplace, leave the hospital grounds, so they can take 15 min every time they smoke a cigarette, which affects their productivity” (Woman, nurse, non-clinician, former smoker)
8: “It generates dysfunction in the work dynamics and although smokers try to go out [to smoke] when the workload is lower, normally they take more time off than they should, affecting the workload of other members of the team” (Woman, doctor, both^a^, former smoker)
Hygiene (13 comments)
9: “The ashes and butts should be cleaned often from the grounds” (Man, doctor, non-clinician, former smoker)
10: “The smoke and smell is noticeable, and can be a bother to the patients and the rest of the staff” (Man, nurse, non-clinician, former smoker)
Tobacco cessation intervention lead by hospital health professionals (8 comments)
11: “I think that helping a smoker to quit is harder than assisting an alcoholic to stop drinking. The person in charge of this must be an expert on this, the rest of health professionals must give a good example and not smoke, at least in public” (Man, doctor, clinician, former smoker)
12: “To assist them (patients) YES, for instance, informing about the harmful effects of smoking, and also to assess their motivation but after this we should refer them to a special unit” (Woman, nurse, both^a^, former smoker)
13: “I consider that our job is to advise patients and support them to quit, at the hospital level too” (Man, nurse, both^a^, former smoker)
14: “A smoker could be more ready to quit smoking in the hospital and if the smoker remains abstinent for some days this could help to start a serious quit attempt” (Woman, nurse, clinician, former smoker)
15: “Tobacco cessation treatment should be provided, after the patient requests” (Women, nurse, clinician, non-smoker)
16: “The National Health System should provide treatment and coordinate tobacco cessation programs better” (Man, nurse, both*former smoker)
Compliance of the new smoke-free law on the hospital grounds (20 comments)
17: “Smoker workers usually comply with the law, but patients and visitors do not…no one corrects their infringements” (Woman, doctor, both^a^, former smoker).
18: “It is very hard for smokers to not smoke outside of the building, but normally the compliance inside the building is correct” (Women, administration, non-clinician, never smoker)
19: “Some people do not understand correctly the non-smoking signs in the hospital campus, despite that the signs posting are clear and at each entrance of the campus” (Man, administration, clinician, former smoker)
20: “Generally the smoke-free campus policy is well complied with, but from time to time we have an infringement in the toilets, that are easy to detect because of the odor” (Women, administration, non-clinician, never smoker)
21: “The infringements have been solved with some verbal reminders. I am not aware of any other penalty” (Woman, nurse, both, never smoker)
22: “The visitors are the ones who infringe the ban most. They smoke at the main entrance of the hospital campus and not outside as posted” (Woman, doctor, both^a^, former smoker)

^a^Clinician and non-clinician

Managers considered that the image and role of hospital workers is a central issue. According to the respondents, smoking in hospitals gives a bad image of the collective and the organization, especially if the ones who break the rule are health workers smoking in their uniforms (see quotes 1, 2, 3 in Table 4). In addition, the lack of cleanliness concerned some of the middle managers who believed that the presence of cigarette butts on the grounds gives a bad example to the community (see quote 3). Some interviewees were also concerned about other hygienic aspects, such as the bad odor given off when smoking.

Ten managers highlighted the detrimental effect on the work dynamics when the smoking policy is not followed. For example, non-smoker employees must take care of the patients of smoker employees and this generates dysfunction in the work dynamic (see quotes 7 and 8).

When asked about tobacco cessation intervention in the hospitals, managers stated its importance, but also the difficulty in giving advice and support to smokers on a daily basis. Respondents considered that general staff should assist smokers, but after brief counsel, smokers should be referred to a special unit or an expert counselor (see quotes 11 and 12). In addition, managers thought tobacco cessation treatments should be available in the hospital and be provided to smokers upon request. Moreover, some respondents mentioned that the National Health System should better coordinate smoking cessation programs and provide such programs free of charge in their service portfolio (see quotes 15, 16).

Finally, when asked about the compliance of the new smoke-free policy, managers felt that smoking workers usually comply with the law; while, patients and visitors were the ones who normally do not adhere to the ban and smoke in illicit places (i.e., in toilets and outdoors). Respondents felt that some visitors and patients do not know about the policy and do not correctly interpret the smoke-free grounds signs posted in the hospital. They suggested applying penalties/fines in cases of infringement. A couple of managers suggested that: “smoker workers must be forbidden to go out to smoke during working hours”.

### Most highly cited terms

**Fig. 1:**
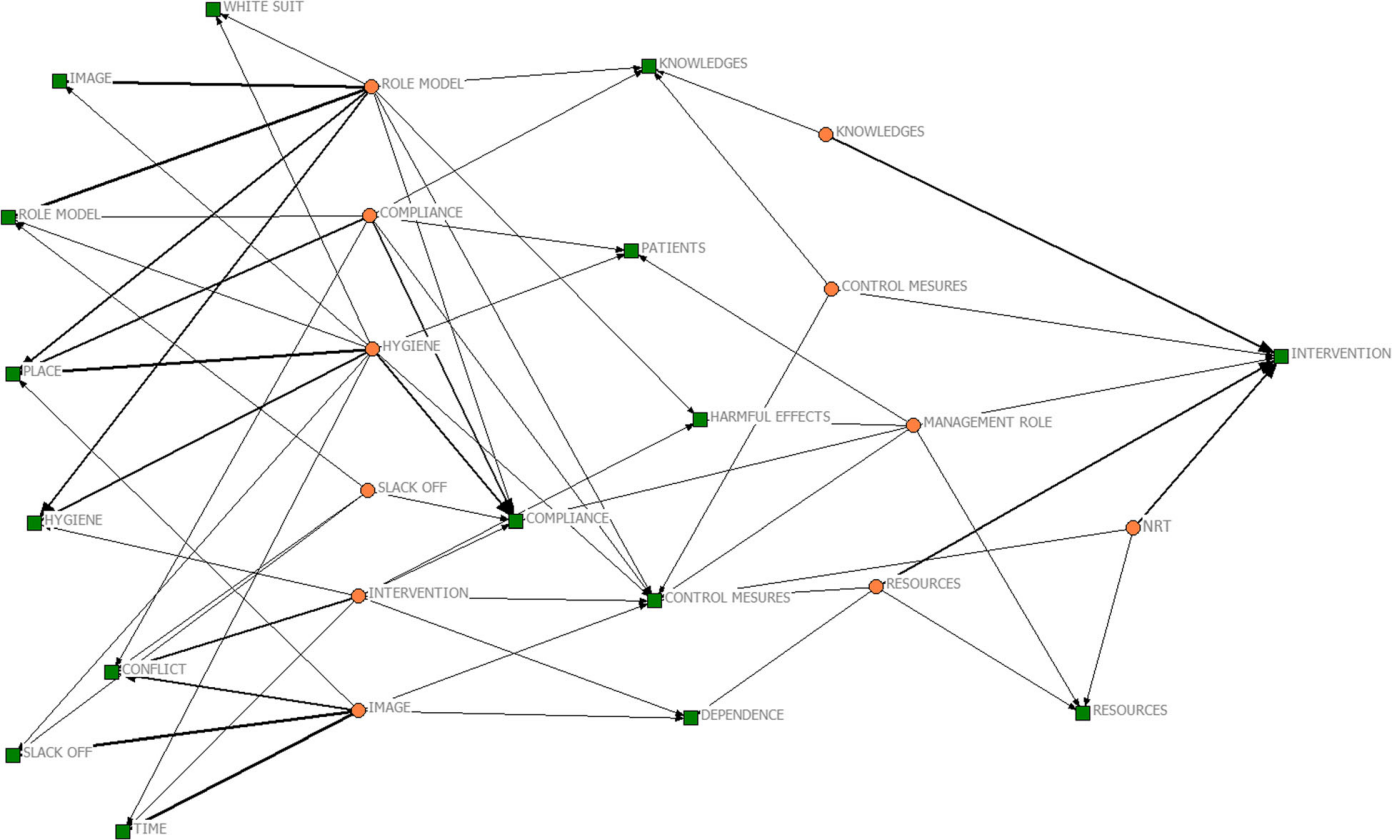
Linkage of terms between question topics (in orange) and answer topics (in green)

Term network (Netdraw ®) used by hospital managers when asked about their open opinion of tobacco control in the hospital is shown in Fig. 1. The terms (orange nodes) used in a high number of responses were role model, image, and hygiene. Furthermore, the most frequent answers (green nodes) were: knowledge, control, intervention, measures, role model, slack off, resources, and nicotine replacement therapy (NRT).

The interrelated connections show that when respondents are asked about role models (orange node) they primarily talked about image, role model, place and hygiene, and secondarily about white suit, compliance, and harmful effects (green nodes). When asked about hygiene (orange node), respondents linked this term primarily with: place, compliance, and hygiene; and secondarily with: white suit, image, role model, time, compliance, and patients. Finally, intervention as a node was related to: conflict, dependence, control measures, hygiene, compliance, and harmful effects.

## Discussion

Our findings revealed several aspects related to middle managers’ (a) behaviors, (b) perceptions, and (c) attitudes after passing a new smoke-free campus policy. First, (a) the behaviors assumed by middle managers were limited and they infrequently reminded others about the policy, encouraged smokers to quit and, solved conflicts related to smoking in the hospital; although with certain reticence in some cases (based on the comments of the participants). In addition, their perception was that tobacco consumption disturbs work dynamics, generates conflicts, threatens the security and hygiene of the hospital, and damages the professional image of health workers. Finally, middle managers showed a supportive attitude towards smoke-free campus policies and unanimously agreed on the role of health providers, managers, and hospitals in tobacco control; although these findings were slightly higher among non-smokers.

Middle managers are purveyors as well as recipients of change. They can sway policy-makers, workers and the public (patients and visitors) [22]. Middle managers should be responsible for enforcing the policy in their respective areas and for addressing policy violations through the existing administrative structure. In our study, we observed that middle managers remind patients of the smoke-free policy more frequently than they remind workers and visitors, but reported having more arguments and conflicts with workers, maybe because it is their role to explain hospital policies to clients and supervise the staff. In the qualitative analysis, we observed that infractions are a frequent topic of concern. In fact, one third of the responders gave their opinion on this regard. In the open responses, middle managers pointed out how the infringements are mainly committed by patients and visitors and only on a few occasions by workers. On the other hand, middle managers recommended quitting more often to smoker workers than to patients. This could be due to the nature of their role as managers of ward(s) and/or unit(s), and their little involvement with patients. Actually, middle managers had different opinions. Some were more prone to think that tobacco cessation interventions should be provided for all hospitalized smokers as part of the hospital’s portfolio and others considered that treatment should be given only when requested by the patients, who should be referred to specialized units. Thus, middle managers’ commitments and active roles in tobacco control are still irregular. These results are in line with other studies that highlight the health providers’ unwillingness to assume the role of imposing and restricting smoke-free policies [15, 23].

According to the “diffusion of innovation model”, the innovations, such as assuming tobacco control policies, should start with the most influential members (supervisors and managers) to non-managerial members (workers). Leaders are in the position to move barriers and change and promote new behaviors [14]. We stated that middle managers may facilitate the implementation of smoke-free campus bans and thereby improve their effectiveness. We observed that the smoking prevalence of middle managers’ (16.2 %) is much lower than that among general health professionals in Catalonia (30.6 %) [6] (Spain). In addition, our data show that middle managers’ smoking status affects how they perceive tobacco consumption. Compared with non-smokers, smokers less often perceived that smoking generates conflicts, which is consistent with other research [15–25], and are more reluctant to think that smoker workers could slack off and affect the exemplary role of health professionals. Thus, middle managers who smoke are less critical of other smokers, especially if the smoker is a hospital worker. This finding suggests that promoting tobacco cessation among middle managers should lead to more proactive behaviors and attitudes towards tobacco control. Nevertheless, smokers had more positive attitudes in regards to providing tobacco cessation treatment, maybe because they are aware of the addictive nature of tobacco and the withdrawal symptoms that smokers experience.

These results can also be understood light of the “Middle managers’ theory” [16]. This theory describes four aspects that mediate middle managers’ commitments during policy implementation in the health care setting. These aspects are: (1) diffusion of information: middle managers disseminate facts, giving employees necessary information about innovation implementation; (2) synthesizing information: middle managers integrate and interpret facts, making general information about innovation implementation relevant to unique organizations and employees; (3) mediating between strategy and day-to-day activities: middle managers identify tasks required for implementing innovations, giving employees the tools necessary to implement them; and (4) selling innovation implementation: middle managers justify innovation implementation, encouraging employees to consistently and effectively use innovations (in this case, follow the new smoke-free campus hospital policy).

Our results showed that middle managers’ commitments are limited and they demonstrate a low level of involvement. Thus, the percentage of those who provide information on the policy is small, only a few support their workers or patients in quitting, and the majority never address day-to-day issues related to smoking in the hospital. However, they express a supportive attitude towards smoke-free campus policies. This difference between attitudes and behaviors could result from deficiencies in other implementation elements that rely on top managers’ responsibilities. This requires further assessment.

Successful implementation of smoke-free policies in healthcare services depends on the previous leadership, organizational culture, communication, training, measurements, and reward systems while creating a decentralized management style and undertaking an end-to-end process view [26]. These can be particularly difficult initiatives for complex organizations, such as those in healthcare. Thus, previous planning, training, and organization are the basic strategies needed for achieving success. The Hospital Consortium of Vic launched a communication campaign 4 months before the law was passed, to inform about the new smoke-free campus rule, and smoke-free billboards and signs were posted. However, middle managers did not receive specific training before the smoke-free ban passage. This could be the reason why some of the respondents had issues about the compliance and their surveillance role.

A smoke-free campus hospital models healthy behavior and gives a clear message that the organization, promotes health, and encourages and facilitates cessation [27]. Moreover, a smoke-free campus leads to a significant reduction in employee smoking [28, 29]. The sociometric and discourse analysis showed that infringements concern middle managers and are related to a bad image (especially when workers are in their uniforms), lack of hygiene, and bad use of work time (interfering with the work dynamic because of the loss of working time). Despite this, middle managers agree on the benefits of implementing a smoke-free hospital campus policy because this projects a role model organization and could help smokers to quit. In addition, they point out the importance of their own role in enforcing the policy; despite their own performances.

There are some limitations to this study. We studied the perceptions, attitudes and behaviors of middle managers, but we were not able to validate or corroborate these data with their real performance. Additionally, the survey was conducted in a single hospital. Middle managers in other hospitals could have different attitudes, likely due to organizational characteristics that vary from hospital to hospital. However, the national law covers all acute hospitals at the same time and a similar hierarchical structure operates in most Spanish hospitals. So, we are inclined to think that similar situations could be found in other Catalan hospitals, as all apply the same policies of the Catalan Network for Smoke-free Hospitals. One of the strengths of our study is the use of both quantitative and qualitative methods to analyze data. By analyzing open-ended answers through content and sociometric techniques, we identified aspects of the smoke-free policy implementation more precisely than using only quantitative methods. Thus, we acquired information about: who the infractors are and why they violate the ban, the opinion of middle managers on providing tobacco cessation in hospitals, the middle managers’ determinants for having a positive or negative opinion on becoming role models in tobacco control, and many other aspects that can only be explored in depth using mixed methods.

## Conclusions

Our findings add to the limited literature examining the role of middle managers in the domain of tobacco control. Our results showed that middle managers agree with the policy, support their own role in making it possible, and perceive the benefits of decreasing smoking by workers, patients and visitors in a hospital where a national tobacco law has been implemented. However, middle managers’ roles could be improved with training, clear protocols for actions in case of infringements, clear descriptions of middle managers’ roles in tobacco control, and the availability of some resources, mainly NRT. Further research is warranted and should include a variety of hospitals with varied geographical, organizational and complexity traits.

## Abbreviations

IQR: Interquartile ranges
NRT: Nicotine replacement therapy
SHS: Second-hand smoke

## References

[1] The Michigan smoke free Campus. Smoke Free Hospitals - The North Carolina Experience. 2007. http://no-smoke.org/index.php. Accessed 09 Sept 2009.

[2] Reid RD, Mullen KA, Slovinec D’Angelo ME, Aitken DA, Papadakis S, Haley PM, McLaughlin CA, Pipe AL (2010). Smoking cessation for hospitalized smokers: an evaluation of the “Ottawa Model”. Nicotine Tob Res.

[3] ENSH (2009). European Network for the Smoke free Hospitals.

[4] Martinez C, Martinez-Sanchez JM, Robinson G, Bethke C, Fernandez E (2014). Protection from secondhand smoke in countries belonging to the WHO European Region: an assessment of legislation. Tob Control.

[5] Fernandez E, Martinez C, Fu M, Martinez-Sanchez JM, Lopez MJ, Invernizzi G, Ouranou A, Dautzenberg B, Nebot M (2009). Second-hand smoke exposure in a sample of European hospitals. Eur Respir J.

[6] Martinez C, Garcia M, Mendez E, Peris M, Fernandez E (2008). Barriers and challenges for tobacco control in a smoke-free hospital. Cancer Nurs.

[7] Knight J, Slattery C, Green S, Porter A, Valentine M, Wolfenden L (2008). Smoke-free hospitals: an opportunity for public health. J Public Health (Oxf).

[8] Schultz AS, Finegan B, Nykiforuk CI, Kvern MA (2011). A qualitative investigation of smoke-free policies on hospital property. CMAJ.

[9] Shopik NA, Schultz AS, Nykiforuk CI, Finegan BA, Kvern MA (2012). Impact of smoke-free hospital grounds policies: patient experiences and perceptions. Health Policy.

[10] Sirkin HL, Keenan P, Jackson A (2005). The hard side of change management. Harv Bus Rev.

[11] Crow G (2006). Diffusion of innovation: the leaders’ role in creating the organizational context for evidence-based practice. Nurs Adm Q.

[12] Tucker AL, Nembhard IM, Edmondson AC (2007). Implementing New Practices: An Empirical Study of Organizational Learning in Hospital Intensive Care Units. Manag Sci.

[13] Birken SA, Lee SY, Weiner BJ, Chin MH, Chiu M, Schaefer CT (2015). From strategy to action: How top managers’ support increases middle managers’ commitment to innovation implementation in health care organizations. Health Care Manage Rev.

[14] Rogers EM (2003). Diffusion of innovations.

[15] Johnson JL, Moffat BM, Malchy LA (2010). In the shadow of a new smoke free policy: A discourse analysis of health care providers’ engagement in tobacco control in community mental health. Int J Ment Health Syst.

[16] Birken SA, Lee SY, Weiner BJ (2012). Uncovering middle managers’ role in healthcare innovation implementation. Implement Sci.

[17] Ballbe M, Nieva G, Mondon S, Pinet C, Bruguera E, Salto E, Fernandez E, Gual A (2012). Smoking and Mental Health Group. Smoke-free policies in psychiatric services: identification of unmet needs. Tob Control.

[18] Campbell S, Pieters K, Mullen KA, Reece R, Reid RD (2011). Examining sustainability in a hospital setting: Case of smoking cessation. Implement Sci.

[19] Martinez C, Fu M, Martinez-Sanchez JM, Ballbe M, Puig M, Garcia M, Carabasa E, Salto E, Fernandez E (2009). Tobacco control policies in hospitals before and after the implementation of a national smoking ban in Catalonia, Spain. BMC Public Health.

[20] Ajzen I (2011). The theory of planned behaviour: reactions and reflections. Psychol Health.

[21] Hughes JR, Keely JP, Niaura RS, Ossip-Klein DJ, Richmond RL, Swan GE (2003). Measures of abstinence in clinical trials: issues and recommendations. Nicotine Tob Res.

[22] Valente TW, Pumpuang P (2007). Identifying opinion leaders to promote behavior change. Health Educ Behav.

[23] Schultz AS, Bottorff JL, Johnson JL (2006). An ethnographic study of tobacco control in hospital settings. Tob Control.

[24] Currie G (1999). The Influence of Middle Managers in the Business Planning Process: A Case Study in the UK NHS. Br J Manage.

[25] Knudsen HK, Studts CR, Studts JL (2012). The implementation of smoking cessation counseling in substance abuse treatment. J Behav Health Serv Res.

[26] Al-Balushi S, Sohal AS, Singh PJ, Al Hajri A, Al Farsi YM, Al AR (2014). Readiness factors for lean implementation in healthcare settings a literature review. J Health Organ Manag.

[27] Sheffer C, Stitzer M, Wheeler JG (2009). Smoke-free medical facility campus legislation: support, resistance, difficulties and cost. Int J Environ Res Public Health.

[28] Wheeler JG, Pulley L, Felix HC, Bursac Z, Siddiqui NJ, Stewart MK, Mays GP, Gauss CH (2007). Impact of a smoke-free hospital campus policy on employee and consumer behavior. Public Health Rep.

[29] Martinez C, Fu M, Martinez-Sanchez JM, Anton L, Fernandez P, Ballbe M, Andres A, Riccobene A, Sureda X, Gallart A, Fernandez E (2014). Impact of a long-term tobacco-free policy at a comprehensive cancer center: a series of cross-sectional surveys. BMC Public Health.

